# Inducible motor neuron differentiation of human induced pluripotent stem cells in vivo

**DOI:** 10.1111/cpr.13319

**Published:** 2022-08-09

**Authors:** Min Chen, Xia Wang, Chuan Li, Ting Lan, Yuhui Wei, Chengcheng Tang, Xiaoqing Zhou, Renping Zhou, Alessandro Rosa, Xi Zheng, Song Ang, Kun Zhang, Qingjian Zou, Liangxue Lai

**Affiliations:** ^1^ Guangdong Provincial Key Laboratory of Large Animal Models for Biomedicine, School of Biotechnology and Health Sciences Wuyi University Jiangmen China; ^2^ International Healthcare Innovation Institute (Jiangmen) Jiangmen China; ^3^ Bioland Laboratory (Guangzhou Regenerative Medicine and Health Guangdong Laboratory) Guangzhou China; ^4^ CAS Key Laboratory of Regenerative Biology, Guangzhou Institutes of Biomedicine and Health Chinese Academy of Sciences Guangzhou China; ^5^ Susan Lehman Cullman Laboratory for Cancer Research, Department of Chemical Biology Ernest Mario School of Pharmacy Piscataway New Jersey USA; ^6^ Department of Biology and Biotechnology Charles Darwin Sapienza University of Rome Rome Italy; ^7^ Center for Life Nano Science Istituto Italiano di Tecnologia Rome Italy; ^8^ Research Unit of Generation of Large Animal Disease Models Chinese Academy of Medical Sciences (2019RU015) Guangzhou China

## Abstract

**Objectives:**

Transplantation of neural progenitor cells (NPCs) derived from human‐induced pluripotent stem cells (hiPSCs) is one of the promising treatment strategies for motor neuron diseases (MNDs). However, the inefficiency in committed differentiation of NPCs in vivo limits its application. Here, we tried to establish a potential therapeutic strategy for MNDs by in vivo directional differentiation of hiPSCs engineered with motor neuron (MN) specific transcription factors and Tet‐On system.

**Materials and Methods:**

We engineered hiPSCs with three MN‐specific transcription factors and Tet‐On system. The engineered cells were directly transplanted into immunodeficient mice through subcutaneous, intra‐spinal cord and intracerebroventricular injections. Following doxycycline (Dox) induction, teratoma formation, and motor MN differentiation were evaluated.

**Results:**

We generated genetically engineered hiPSCs, in which the expression of *Ngn2*, *Isl1*, and *Lhx3* was controlled by a drug‐inducible transgenic system. These cells showed normal pluripotency and proliferative capacity, and were able to directionally differentiate into mature motor neurons (MNs) and NPCs with high efficiency in spinal cords and cerebral lateral ventricles under the induction of Dox. The grafts showed long‐term survival in the recipient mice without formation of teratoma.

**Conclusions:**

The induced mature MNs and NPCs were expected to replace the damaged endogenous MNs directly, and play a role of de novo stem cell stock for long‐term neuron damage repair, respectively. Therefore, in vivo directional differentiation of the hiPSCs engineered with MN‐specific transcription factors and Tet‐On system via Dox induction could be a potential therapeutic strategy for MNDs with high efficacy and safety.

## Graphical abstract

Generation of hiPSCs with multi‐gene modifications, including motor neuron‐specific transcription factors *Ngn2*, *Isl1*, and *Lhx3* controlled by Tet‐On system and one anti‐apoptosis gene *Bcl‐xL*. The genetically modified hiPSCs survived and directionally differentiated into motor neurons after transplantation in vivo*.* The genetically modified hiPSCs partially converted into neural progenitors, serving as a spare cell resource.

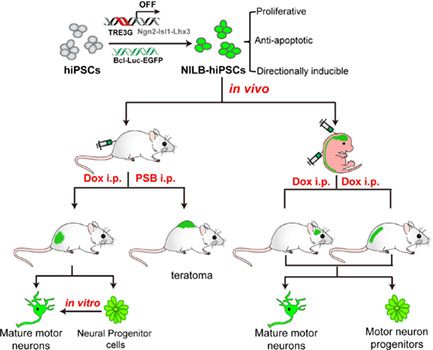

## 1 INTRODUCTION

Selective motor neuron loss is the most common and striking pathology observed in motor neuron diseases (MNDs), such as amyotrophic lateral sclerosis (ALS) and spinal muscular atrophy. Owing to the difficulty of neuronal regeneration, clinical studies have failed to develop effective strategies to replace lost motor neurons (MNs). Stem cells have great potential for the replenishment of lost neuronal population in MNDs. Intensive efforts have been made in developing stem cell‐based strategies for MNDs. In particular, mesenchymal stem cells are being tested in early‐stage clinical trials due to their good safety and tolerability. However, mesenchymal stem cells cannot differentiate into neurons and have only shown limited beneficial effects in clinical studies.^1^, ^2^, ^3^ Recently, cell transplantation studies have shifted to neural progenitor cells (NPCs) obtained from embryonic tissues or pluripotent cells. NPCs can self‐renew and differentiate into astrocytes, oligodendrocytes, and neurons to build neural networks.^4^ However, these cells are likely to differentiate into glial cells rather than into functional neurons, which is a disadvantage for neuron replacement therapy.^5^ As a solution, neuronal restricted progenitors and motor neuron progenitors (MNPs) derived from human embryonic stem cells (hESCs)/human‐induced pluripotent stem cells (hiPSCs) have been engineered and developed into neurons rather than glial cells or other cell types in vivo and in vitro.^6^, ^7^ However, the purity and differentiation potential of these neural cells are substantially diminished during cell passage.^8^ Although hESC/hiPSC‐derived MNs can replace the lost neuronal population directly without glial formation, the terminally differentiated neurons are source limited, fragile, and likely to die after transplantation.^9^

The in situ direct conversion of endogenous astrocytes to functional neurons by the ectopic expression of defined factors^10^ or the knockdown of RNA‐binding protein PTB in vivo is another therapeutic strategy for neurodegenerative disorders.^11^ However, the potential adverse effects caused by local astrocyte depletion and microenvironment alteration are unknown. Neurodegenerative diseases are often accompanied by gene mutations that can hardly be repaired by in situ conversion, resulting in the failure of disrupted circuit reconstruction. If the injury or degeneration is severe, then in vivo cell conversion might not be sufficient to generate enough cells. Exogenous functional cells or artificial tissues can provide a rich source to repair tissue loss.^12^ Neural‐specific genes can convert engineered human fibroblasts and astrocytes into neurons in vivo^10^; however, the conversion efficiency is extremely low (0.4%–5.9%), resulting in limited cell regeneration.

Three MNs that induce transcription factors, namely, *Ngn2*, *Isl1*, and *Lhx3*, can efficiently induce functional MNs with mature electrophysiological properties from hESCs or hiPSCs.^13^, ^14^A previous preclinical study differentiated MNs in vitro and then transferred them in animal models to treat MNDs.^15^ However, the insufficient number and poor survival of terminal MNs after transplantation limit the application of this approach in stem cell therapy. We hypothesized that these two limitations can be overcome by directly transferring engineered hiPSCs, which are easily available in a large number and have good survival capacity, into recipients and then inducing them into MNs in vivo rather than in vitro. To test this concept, we generated genetically engineered hiPSCs and incorporated them in a drug‐inducible transgenic system to control the expression of *Ngn2, Isl1*, and *Lhx3* genes. The cells showed normal proliferative capacity and directional differentiation into MNs in doxycycline (Dox) medium in vitro. Further in vivo experiments revealed that these engineered hiPSCs could convert into MNs and NPCs whether in subcutaneous tissues, spinal cords, and cerebral lateral ventricles. These induced mature MNs may be used to directly replace the damaged endogenous MNs, and NPCs may provide de novo stem cell stock for long‐term neuron damage repair.

## 2 MATERIALS AND METHODS

### 2.1 Plasmid construction

The PB‐*Ngn2‐Isl1‐Lhx3‐BSD* (PB‐*NIL*) plasmid was described in De Santis et al.^16^ The PB‐*Bcl‐xL‐Luciferase‐GFP* (PB‐*BLG*) plasmid was generated by inserting the sequences of *Bcl‐xL* (gene ID 397536) and firefly luciferase‐GFP (LG, pGL4.21) in the PB vector. pGL4.21 was purchased from Promega.

### 2.2 Cell experiments

HiPSCs were co‐transfected with transposable vectors and PiggyBac transposase (PB‐*NIL*: transposase = 4:1 or PB‐*NIL*: PB‐*BLG*: transposase = 2:2:1) using Lipofectamine LTX (Invitrogen). After blasticidin (BSD, 10 μg/ml, Invitrogen) selection, the BSD‐positive colonies were collected with a pipette and used to generate two stable hiPSC lines (termed *NIL*‐hiPSCs and *NILB*‐hiPSCs). MN differentiation of *NIL*‐hiPSCs and *NILB*‐hiPSCs was induced by the addition of 1 μg/ml Dox (Sigma) in MN induction medium.^16^ After 5 days, these induced MNs were maintained in the MN medium. After 4 weeks, the cells were dissociated with Accutase (Stem Cell) and plated on Matrigel (Corning)‐coated wells. Neural cells were expanded in the MN medium.

For further differentiation, the culture medium was switched to the MN induction medium. (i) In vitro differentiation was evaluated by immunofluorescence staining using MN, NPC, and MNP markers; (ii) the functional characteristics of hiPSC‐derived MNs were determined by patch‐clamp recordings; and (iii) the pro‐survival capacity of *Bcl‐xL* was determined by replating‐induced stress^17^ and glutamate toxicity assay.^18^

### 2.3 Animal experiments

All animal care and experimental procedures were approved by the Ethical Committee on Animal Experiments at Guangzhou Institutes of Biomedicine and Health, Chinese Academy of Sciences. *NILB*‐hiPSCs were directly transplanted into immunodeficient mice without in vitro differentiation through subcutaneous, intra‐spinal cord, or intracerebroventricular injections. Following Dox induction (25 mg/kg, i.p., five times), teratoma formation and MN differentiation were evaluated as follows: (i) the survival of transplanted cells was tracked by bioluminescent imaging; (ii) tumorigenicity was determined by the haematoxylin and eosin (H&E) staining of three germ layers and expression of pluripotent markers; (iii) in vivo differentiation was evaluated by immunofluorescence staining using MN, NPC, and MNP markers; and (iv) the functional characteristics of hiPSC‐derived MNs were determined by brain and spinal cord slice electrophysiology.

### 2.4 Statistical analysis

All statistics, including statistical tests, sample sizes, and types of replicates, were described in the figure legends. A *p* value of <0.05 was considered statistically significant.

Detailed experimental procedures are described in the Supporting Information Material S1.

## 3 RESULTS

### 3.1 Establishment of multi‐gene‐modified hiPSCs

**FIGURE 1:**
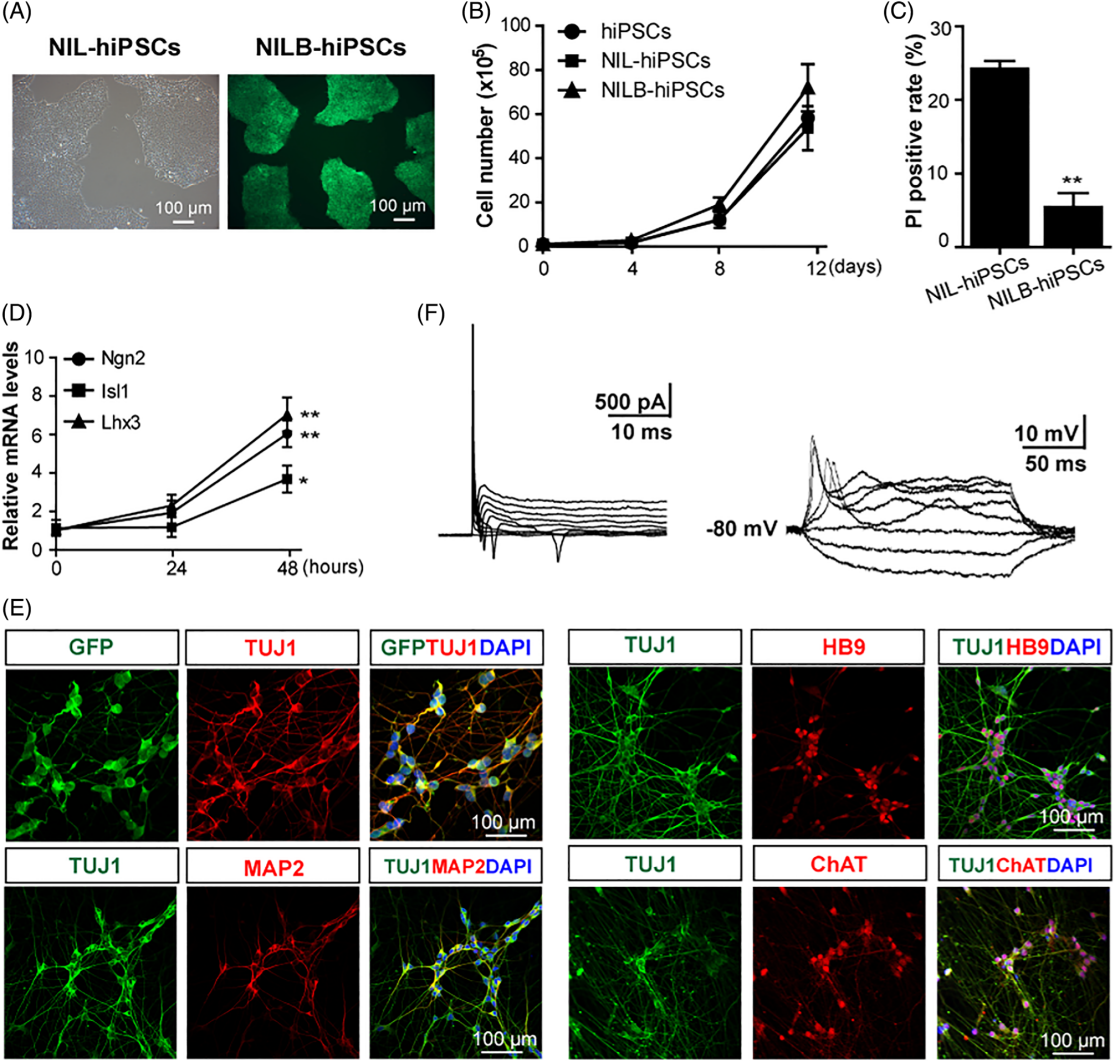
Establishment of multi‐gene‐modified hiPSCs. (A) Cell colony morphology of *NIL*‐hiPSCs and *NILB*‐hiPSCs, bar = 100 μm. (B) The proliferation curve of hiPSCs, *NIL*‐hiPSCs, and *NILB*‐hiPSCs during in vitro culture (*n* = 3, each group; error bars represent SEM). (C) Apoptosis analysis of *NIL*‐hiPSCs and *NILB*‐hiPSCs by PI staining at 3 days after dissociation (*n* = 3, each group; error bars represent SEM; two‐tailed Student's *t* test, **p < 0.01). (D) RT‐qPCR analysis of inducible expressed *Ngn2*, *Isl1*, and *Lhx3* at the indicated time points after Dox treatment (*n* = 3, each group; error bars represent SEM; two‐tailed Student's *t* test, *p < 0.05, **p < 0.01). (E) Immunostaining for neuronal markers TUJ1 and MAP2, MNs markers HB9 and ChAT, bar = 100 μm. (F) Representative traces of action potentials and voltage‐dependent ion currents recorded in *NILB*‐hiPSCs‐derived MNs. See also Figure S1

The vector PB‐*NIL* contains Dox‐inducible polycistronic genes *Ngn2, Isl1*, and *Lhx3* (*NIL*) and constitutively expresses BSD resistance gene (Figure S1A). The other vector named PB‐*BLG* contains an anti‐apoptotic gene *Bcl‐xL* and two tracker genes, luciferase and green fluorescent protein (EGFP, Figure S1A). These vectors were transfected into hiPSCs to generate two stable hiPSCs, namely *NIL*‐hiPSCs (containing only PB‐*NIL*) and *NILB*‐hiPSCs (containing PB‐*NIL* and PB‐*BLG*). Both cell lines formed round colonies with clear margins similar to those of normal hiPSCs, and *NILB*‐hiPSCs were positive for EGFP (Figure 1A). For proliferative capacity, no statistical difference was observed among *NIL*‐hiPSCs, *NILB*‐hiPSCs, and parental hiPSCs (Figure 1B), indicating that both engineered hiPSC lines retained normal proliferative capacity in mTeSR1 medium. Propidium iodide (PI) was used to stain the dead cells after passage in vitro. The PI‐positive proportion of *NILB*‐hiPSCs (5.43% ± 1.90%) was markedly lower than that of *NIL*‐hiPSCs (24.22% ± 1.10%, Figures 1C and S1B).

### 3.2 Validation of the directional differentiation of multi‐gene‐modified hiPSCs in vitro

Dox concentrations from 0 to 1 μg/ml in the medium were used to induce *NILB*‐hiPSC differentiation. No cells changed phenotype without Dox treatment, and all cells differentiated to neuron‐like cells under 1 μg/ml Dox (Figures S1C–E). Thus, 1.0 μg/ml Dox was used for the following experiments. RT‐qPCR test results showed that the expression levels of *Ngn2*, *Isl1*, and *Lhx3* increased significantly 48 h after Dox treatment (Figure 1D). Similarly, up to 90% of *NILB*‐hiPSCs expressed ectopic *Isl1* according to the immunofluorescence test (Figure S1F). These indicated that the expression of MN transcription factors can be tightly controlled by Dox.

Whether Dox treatment could directly induce MNs from *NILB*‐hiPSCs was investigated. After treatment with Dox for 5 days, neuron‐like cells with condensed nuclei, long axons, and multiple neurites were formed. These induced MNs stained positive for pan‐neuronal markers including TUJ1 and MAP2 and motor neuronal marker HB9. When the cells were further cultured to 14 days, ChAT, a marker of mature neurons, was also detected (Figure 1E). Statistical data showed that 94.66% ± 2.02% differentiated cells were positive for TUJ1, in which the percentages of MAP2‐, HB9‐, and ChAT‐positive cells were 81.64% ± 2.44%, 83.93% ± 3.70%, and 89.15% ± 2.26%, respectively (Figure S1G). After 4 weeks, whole‐cell patch‐clamp recordings were performed to further characterize the functional membrane properties of the induced MNs. The neurons generated repetitive traces of action potentials as observed in the current clamp records (Figure 1F). The voltage clamp records showed rapidly inactivating inward currents (Na^+^) and persistent outward currents (K^+^) in response to depolarizing voltage (Figure 1F). All these data indicated that *NILB*‐hiPSCs had efficiently differentiated into electrophysiologically mature MNs in vitro.

**FIGURE 2:**
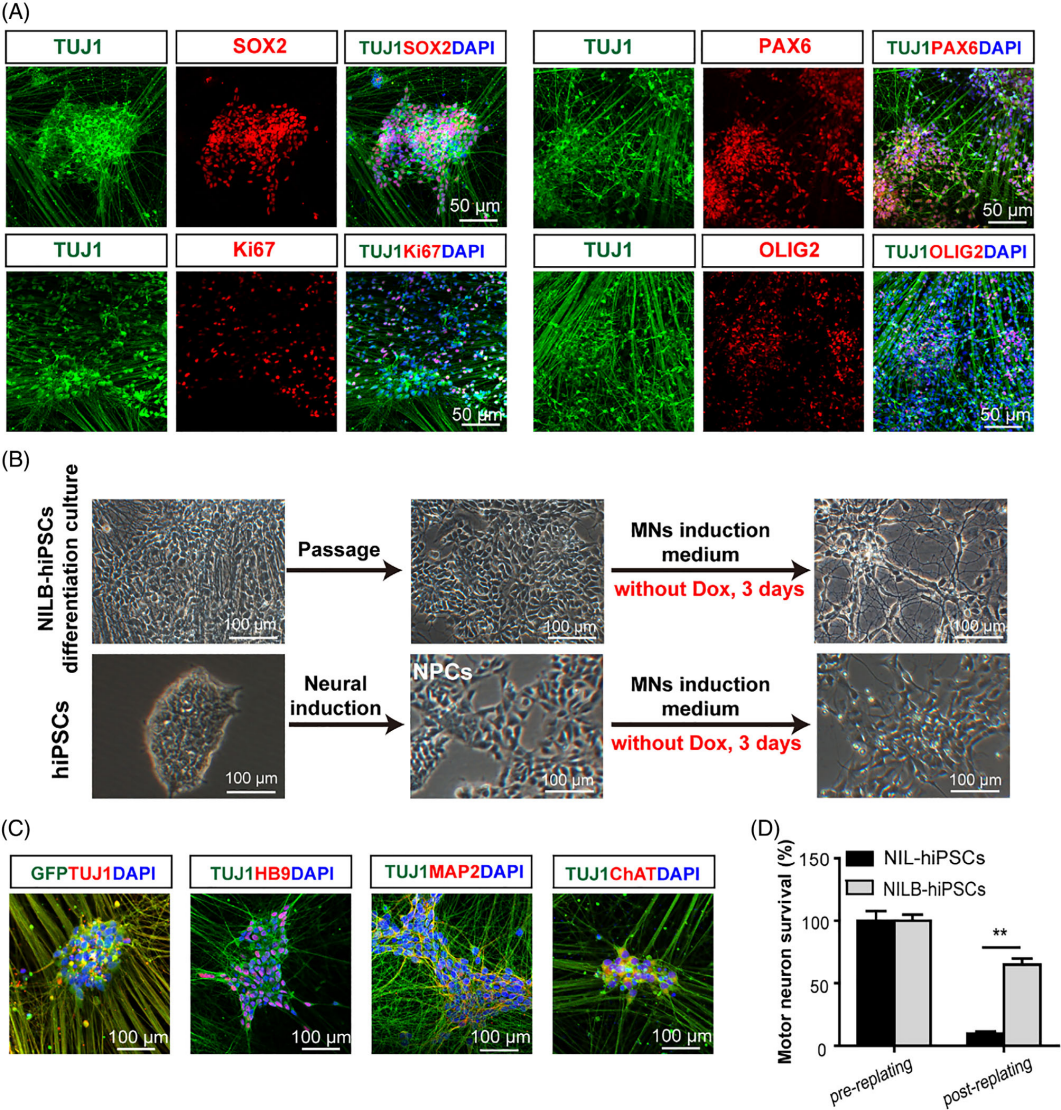
Long‐time differentiation of multi‐gene‐modified hiPSCs. (A) Progenitor markers SOX2, PAX6, Ki67, and OLIG2 in the prolonged *NILB*‐hiPSCs differentiation culture. (B) Schematic diagram of re‐differentiation of induced NPCs derived from *NILB*‐hiPSCs. Upper panel: *NILB*‐hiPSCs differentiation culture were passaged and cultured in MNs induction medium, bar = 100 μm; lower panel: hiPSCs‐induced NPCs were cultured in the same medium, bar = 100 μm. (C) Neuronal markers expression in re‐differentiated MNs at indicated time points, bar = 100 μm. (D) Replating‐induced stress model to verify the pro‐survival capacity of *NILB*‐hiPSCs‐derived MNs (*n* = 3, each group; error bars represent SEM; two‐tailed Student's *t* test, **p < 0.01). See also Figure S2

When *NILB*‐hiPSC differentiation culture was prolonged beyond 2 weeks, non‐neuronal‐like cells emerged and proliferated among the mature MNs (Figure S2A). This cell cluster expressed NPC markers SOX2 and PAX6, and proliferating marker Ki67 at 3 weeks. At 4 weeks of differentiation, many progenitor cells expressed MNP marker OLIG2 (Figure 2A). The cultures were dissociated into single cells and expanded in the MN medium to obtain these progenitors (Figure 2B). The cells showed a strong tendency to differentiate into TUJ1‐positive cells in a rapid (3 days) and effective (93.86% ± 0.93%) manner when cultured in MN induction medium without Dox administration. Meanwhile, neuron‐like cells were formed by only 49.09% ± 1.85% of the cells that differentiated from ordinary human NPCs (Figures 2C and S2B,C). These neuron‐like cells were positive for MAP2, HB9, and ChAT (Figure 2C) with ratios of 83.10% ± 2.88%, 89.08% ± 0.70%, and 83.75% ± 1.94%, respectively (Figure S2D). Furthermore, the MN differentiation of *NILB*‐hiPSCs was tested in a 3D cell culture, and similar outcomes were observed: by the 4th week of differentiation, the spheroids showed a large size, a high cell number, and robust expression of SOX2, PAX6, OLIG2, and Ki67 (Figure S2E).

Two different in vitro models were used to determine the pro‐survival capacity of *Bcl‐xL*: replating‐induced stress^17^ and glutamate excitotoxicity.^18^ In the first model, 64.93% ± 8.47% of MNs from *NILB*‐hiPSCs survived, which was higher compared with the 9.94% ± 2.68% of MNs from *NIL*‐hiPSCs after passage in vitro (Figure 2D, *p* < 0.01). The second experiment showed that the MNs from *NIL*‐hiPSCs had great susceptibility to glutamate, with only 46.43% ± 9.45% of the induced MNs surviving after glutamate treatment; meanwhile, up to 73.26% ± 7.60% of the neurons from *NILB*‐hiPSCs survived (Figure S2F, *p* < 0.05). The ectopic expression of *Bcl‐xL* conferred the pro‐survival ability of MNs, suggesting that induced MNs can survive and perform their function after transplantation in vivo. Thus, *NILB*‐hiPSCs were used for the following in vivo experiments.

### 3.3 In vivo induction of subcutaneously injected *NILB*‐hiPSCs

**FIGURE 3:**
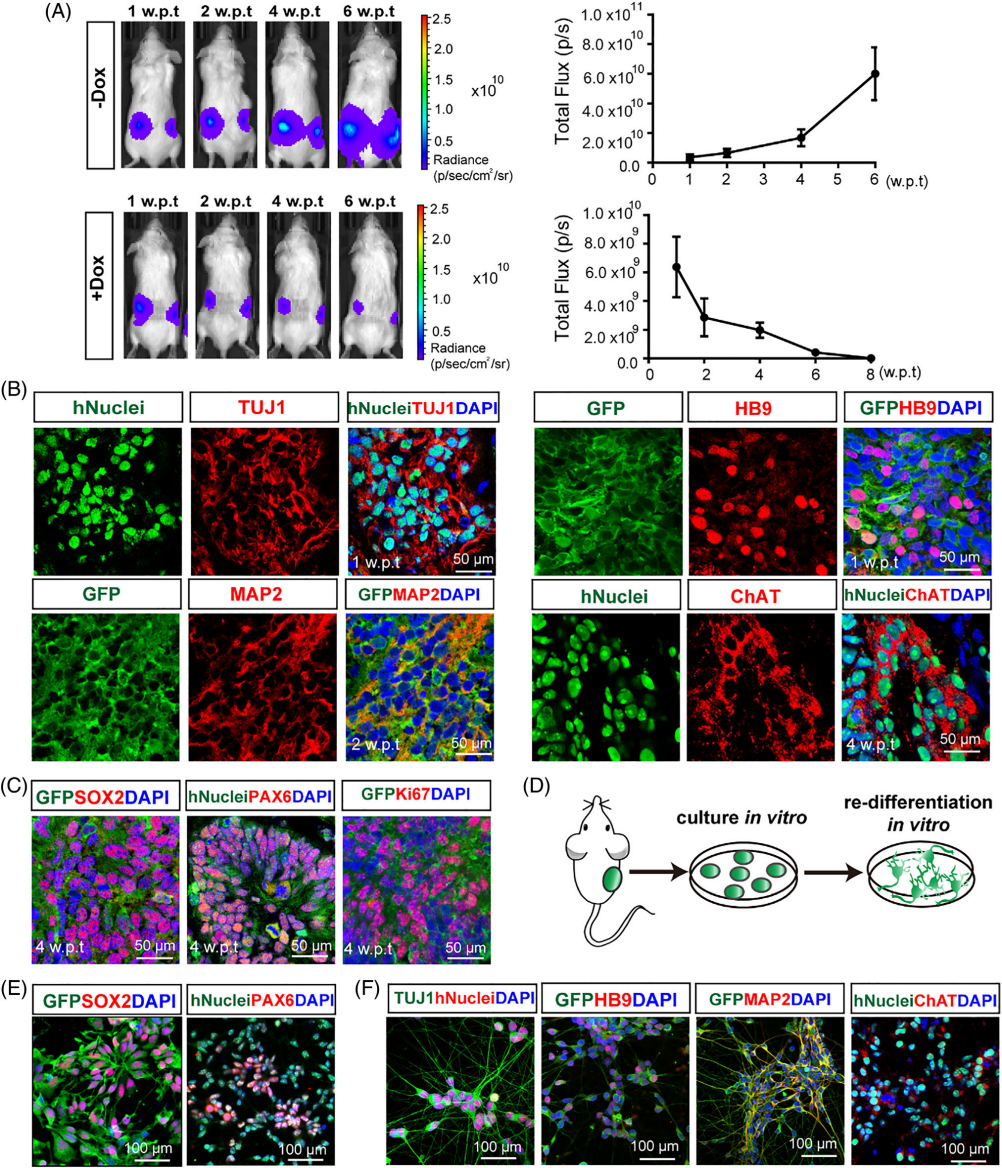
In vivo induction of subcutaneously injected *NILB*‐hiPSCs. (A) Upper Panel: In vivo imaging of luciferase activity after subcutaneous transplantation in untreated control group (*n* = 4, each group; Error bars represent SEM). Lower Panel: In vivo imaging of luciferase activity after subcutaneous transplantation in Dox‐treated group (*n* = 4, each group; Error bars represent SEM). (B) Immunostaining for neuronal markers TUJ1, MAP2, HB9 and ChAT in *NILB*‐hiPSCs‐transplanted animals, bar = 50 μm. (C) Immunostaining for NPCs markers SOX2, PAX6 and proliferating marker Ki67 in 4‐week of *NILB*‐hiPSCs‐transplanted animals, bar = 50 μm. (D) Schematic diagram of re‐differentiation of grafts in MNs induction medium. (E) NPCs markers SOX2 and PAX6 expression in the engraftments cultured in vitro, bar = 100 μm. (F) Neuronal markers expression in the engraftments‐derived MNs at indicated time points, bar = 100 μm. See also Figure S3

*NILB*‐hiPSCs were subcutaneously injected into immunodeficient mice to initially evaluate the differentiation direction of these engineered hiPSCs in vivo. In vivo imaging showed that the luciferase activity increased rapidly with time in the control group (without treatment of Dox) (Figure 3A, upper panel), indicating that the uninduced *NILB*‐hiPSCs proliferated dramatically in the transplanted sites. Upon Dox treatment (25 mg/kg/day, i.p.) for 5 days, the luciferase activity decreased in a time‐dependent manner, was almost undetectable at 6 weeks, and completely disappeared at 8 weeks post‐transplantation (w.p.t.) (Figure 3A, lower panel). Engraftments were retrieved at different time points (1, 2, 4, and 6 w.p.t.). The *NILB*‐hiPSCs in the untreated control group formed a teratoma‐like structure with increasing size over time, and those in the Dox‐treated group showed a white and loose engraftment at 2 w.p.t., that became small and compacted at 4 w.p.t. and further shrunk at 6 w.p.t. (Figure S3A). Histological test by H&E showed that the tissues from the untreated group exhibited the typical structures of tri‐germ layers at 6 w.p.t. (Figure S3B, left panel). For the treated group, the structures of tri‐germ layers were not discerned, but a pyramid neuron‐like structure was observed at 2 w.p.t., and massive ventricle‐like cavities were formed at 4 w.p.t. (Figure S3B, right panel). The tissues from the treated group were further evaluated by immunofluorescence staining to determine the differentiation status of the transplanted cells. At 1 w.p.t., most of the cells were human nuclear antigen positive (hNuclei^+^), that is, they were transplanted human cells. Many of the human cells expressed TUJ1 (68.93% ± 9.61%; Figures 3B and S3C). Further test with neuronal subtype marker showed that GFP^+^ cells, which were *NILB*‐hiPSCs marked with GFP, expressed MAP2 and HB9 (MAP2^+^/GFP^+^, 61.43% ± 6.15%; HB9^+^/GFP^+^, 43.89% ± 3.24%, Figures 3B and S3C). At 4 w.p.t., many human cells expressed mature motoneuronal marker ChAT (57.64% ± 8.05%, Figures 3B and S3C), suggesting that the transplanted *NILB‐*hiPSCs can be directly converted to mature MNs in vivo after Dox induction.

Massive ventricle‐like cavities composed of hNuclei^+^ cells appeared in the tissues at 4 w.p.t. (Figure S3D). These cells expressed SOX2 and PAX6, two NPC markers, and Ki67, a proliferating marker (Figure 3C). The grafts (4 w.p.t.) were further isolated and cultured in vitro (Figure 3D). The derived primary cells from these grafts could proliferate and form rosette‐like structures in vivo and express SOX2 and PAX6 (Figure 3E) but not OLIG2 (Figure S3E). They rapidly and effectively re‐differentiated into MNs in the MN induction medium (Figures 3F and S3F). When cultured in non‐MN induction medium supplemented with fetal bovine serum, about 64.83% ± 5.33% of the derived primary cells differentiated into GFAP‐positive glia cells (Figure S3G). These data indicated that some *NILB*‐hiPSCs formed NPCs in subcutaneous segment, and these induced NPCs can give rise to neuronal and glial cells.

### 3.4 In vivo induction of *NILB*‐hiPSCs after intra‐spinal cord and intracerebroventricular injections

**FIGURE 4:**
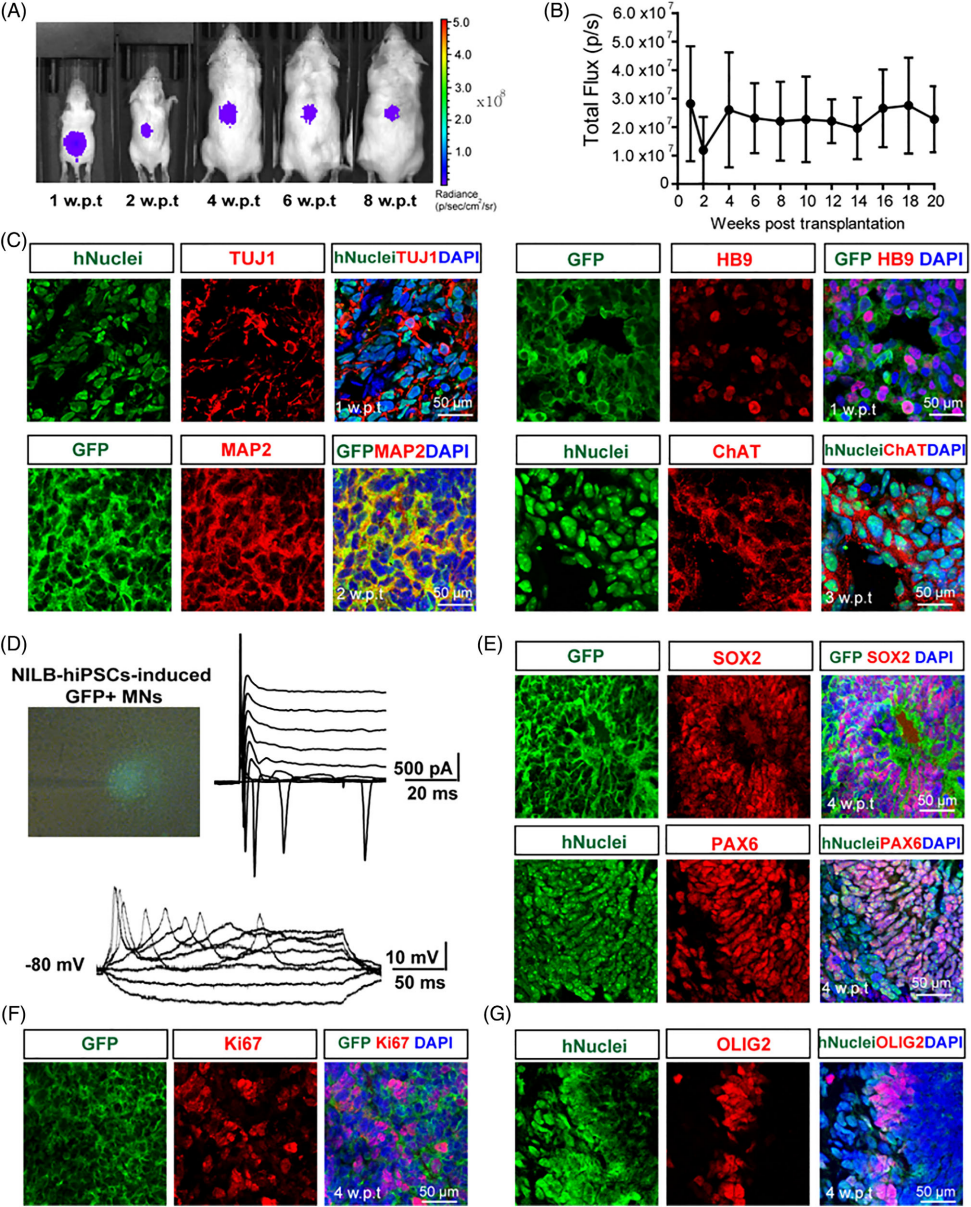
In vivo induction of *NILB*‐hiPSCs after intra‐spinal cord injections. (A) Imaging of luciferase activity in spinal cords of *NILB*‐hiPSCs‐transplanted mice. (B) Luciferase signal curve of grafts changed over time (*n* = 3, each group; error bars represent SEM). (C) Immunostaining for neuronal markers TUJ1 and MAP2, HB9 and ChAT in the spinal cords of *NILB*‐hiPSCs‐transplanted animals, bar = 50 μm. (D) Representative traces of action potentials and voltage‐dependent ion currents recorded in *NILB*‐hiPSCs‐derived MNs after intra‐spinal cord transplantation. (E) Immunostaining for NPCs markers SOX2 and PAX6 in the spinal cords of *NILB*‐hiPSCs‐transplanted animals, bar = 50 μm. (F) Immunostaining for proliferating marker Ki67 in the spinal cords of *NILB*‐hiPSCs‐transplanted animals, bar = 50 μm. (G) Immunostaining for OLIG2 in the spinal cords of *NILB*‐hiPSCs‐transplanted animals, bar = 50 μm. See also Figure S4 and S5

Age‐related decline of neurogenic niche in the brain,^19^ as well as decreased migration capacity of grafts along with age of the recipient animals have been reported previously.^20^, ^21^ Therefore, neonatal mice (postnatal day 4) were chosen to test effects of in vivo induction after the engineered hiPSCs were transplanted into the spinal cords and cerebral ventricles. The luciferase signal was markedly reduced at 2 w.p.t., but bounced back at 4 w.p.t., and remained stable from 4 to 20 w.p.t. (Figures 4A,B and S4A,B). Immunofluorescence staining revealed that in the brain of mice at 2 w.p.t., the injected *NILB*‐hiPSC‐derived cells were dispersed throughout the cerebral ventricles and formed extensive dendritic arborizations (Figure S4C). At 2 w.p.t., the vast majority of hNuclei^+^ cells in mice (68.81% ± 3.53% in spinal cords; 75.70% ± 2.88% in cerebral ventricles) were positive for the neuronal marker TUJ1 (Figures 4C and S4D,E), indicating the predominant differentiation of transplanted *NILB*‐hiPSCs to neurons in the central nervous system (CNS). Further immunostaining showed that the grafts expressed mature MN markers, including MAP2 (57.56% ± 4.58% in spinal cords; 54.67% ± 11.21% in cerebral ventricles), HB9 (45.43% ± 9.75% in spinal cords; 40.26% ± 2.16% in cerebral ventricles), and ChAT (59.92% ± 8.72% in spinal cords; 62.81% ± 14.50% in cerebral ventricles; Figures 4C and S4D,E). Moreover, the electrophysiological characteristics of transplanted cells were examined at 4 w.p.t. Patch‐clamp recording in GFP^+^ neurons showed the robust trains of action potentials and large voltage‐gated sodium/potassium currents (Figures 4D and S4F), suggesting that the transplanted *NILB*‐hiPSCs can be directionally converted to electrophysiologically mature MNs in the CNS after Dox induction.

Rosette‐like structures and ventricle‐like cavities were also observed in the brain and spinal cord cryosections of mice at 4 w.p.t. (Figures 4E and S5A). Proliferating marker Ki67^+^ was also intermixed with the cells expressing SOX2 and PAX6 (Figures 4F and S5A), and this finding was consistent with the bioluminescent imaging data above (Figures 4A,B and S4A,B). Unexpectedly, a small portion of transplanted cells expressed OLIG2, suggesting the presence of MNPs (Figures 4G and S5A). None of the grafts remained pluripotent (negative for NANOG) or formed teratomas (Figure S5B). All these data indicated that the transplanted cells can differentiate into MNs, NPCs, and MNPs in the CNS after Dox treatment and the differentiated cells could survive for a long term.

## 4 DISCUSSION

In this study, we engineered hiPSCs with the three MN transcription factors *Ngn2, Isl1*, and *Lhx3* and Tet‐On system. The engineered cells could directionally and efficiently differentiate into MNs and NPCs in vitro and in vivo by Dox induction.

*Ngn2*, *Isl1*, and *Lhx3* can directionally induce pluripotent cells and fibroblasts into mature MNs.^16^, ^17^ This method can bypass the neural progenitor stage, which has been observed in conventional multi‐step differentiation techniques using small molecules.^22^, ^23^ In our inducing system, neuron‐like cells with condensed nuclei, long axons, and multiple neurites were formed at 5 days after induction and expressed neuronal markers, including TUJ1, MAP2, and HB9. With prolonged culture time, these neurons turned into mature MNs with ChAT expression and typical electrophysiological properties. These data indicated that *NILB*‐hiPSCs could be efficiently induced into mature MNs by Dox. *NILB*‐hiPSCs differentiated into MAP^+^ neurons at 2 w.p.t. and into ChAT^+^ and functionally mature MNs in vivo at 3–4 weeks. This duration was faster than that in previous observations using hiPSC‐derived NPCs. Preclinical studies documented that after transplantation in vivo, hiPSC‐derived primitive NPCs need 1 month to form DCX^+^ immature neurons and at least 2 months to form MAP^+^ mature neurons in ALS rats.^24^, ^25^ The MNs induced by our strategy can rapidly replace the damaged endogenous MNs, which is particularly an advantage in curing acute spinal cord injury.

In this work, *NILB*‐hiPSCs had differentiated not only into mature MNs but also to NPCs in vitro and in vivo; this phenomenon was not observed in previous studies.^16^, ^17^ Previous dynamics studies showed that *Ngn2* plays a crucial role in the outcomes of neural differentiation: *Ngn2* induces functional neurons when its expression is sustained but induces NPCs when its expression oscillates.^26^ However, the regulating mechanism for oscillatory versus sustained *Ngn2* expression remains to be determined. A recent study showed that *Ngn2*‐modified messenger RNA (mmRNA) can simultaneously program hiPSCs into neurons and NPCs due to the intrinsic fluctuations in mmRNA and protein levels.^27^ In our inducing system, most cells expressed a sufficient amount of *Ngn2* to directly convert *NILB*‐hiPSCs into MNs under Dox induction. However, a small number of *NILB*‐hiPSCs might express a low level of *Ngn2* with oscillatory‐like pattern under Dox induction, thus resulting in NPC formation. Brain development studies showed that neurogenesis declines with ageing.^28^ Only small portion of NPCs exist in the adult brain and are restricted in the hippocampus and striatum. They remain in a rest state and will be activated to proliferate, migrate, and differentiate to replace lost neurons after an injury.^29^ However, these NPCs have an extremely limited capability for regeneration and thus cannot effectively replace injured neurons. The de novo NPCs derived from *NILB*‐hiPSCs could survive long‐term in vivo and may play the role of stem cell backup to fix injured neurons. This characteristic should be a potential advantage in curing chronical neurodegenerative diseases, such as ALS.

HiPSCs have a tendency to form a teratoma, which is the main concern of clinical practices. The generated *NILB*‐hiPSCs also developed a teratoma after their subcutaneous transplantation without Dox treatment. However, after Dox treatment for only 5 days, the destination was narrowed to neural cell lineage, and no tumour formation was noted in the subcutaneous tissues. The induced MNs gradually dwindled and ultimately disappeared at 8 w.p.t., which differed from the situation in the CNS. We speculate that this phenomenon probably occurred because the neurons could not adapt to the non‐neural microenvironment when transplanted into the subcutaneous tissues. Our results confirmed that the induced MNs survived well in the CNS for more than 20 weeks. The microenvironmental conditions of the transplant site may contribute to cell survival and function. Most of the transplanted cells initially died after being transplanted into the pathological environment. Pro‐survival strategies have been reported, including genetic modifications, cell preconditioning, and use of biomaterials.^4^ Among them, genetic modifications can enable the stem cells for anti‐immune rejection, anti‐inflammation, and increasing angiogenesis should be conducted prior to the transplantation to enhance their survival capacity.^30^

Despite these beneficial potentials, the safety risks of engineered pluripotent cells are still the most important challenge for their clinical application. Overdose of Dox treatment may lead to side effects on gastrointestinal tract,^31^ while the insufficient induction caused by the inaccessibility of Dox to some cells will make grafts out of control, resulting in teratoma formation. In addition, the insertion of a foreign gene in an inappropriate site in the stem cells might give rise to tumorigenesis. Therefore, before human clinical trials, it is necessary to conduct experiments on the large animal models, such as pigs, dogs and monkeys to determine the optimized dosage and administration route. Besides, inducible suicide systems could be introduced into hiPSCs to reduce the risk of uncontrollable pluripotent cells by killing the grafted tumorigenic cells^32^ or the undifferentiated cells.^33^

In summary, we describe a rapid, effective, and long‐lasting approach that generates an enriched population of MNs in vivo and should be valuable for the treatment of MNDs. Further studies are required to determine the therapeutic effects of these hiPSCs on small and large animal models of MNDs. A previous study showed that hiPSCs could be directly converted into a variety of specific human cells under the action of lineage‐specific transcription factors.^34^ If *Ngn2*, *Isl1*, and *Lhx3* factors in our system are replaced by other cell type‐specific transcription factors, then diverse cell types could be induced in vivo to treat corresponding organ failure or tissue loss. From this point of view, the developed novel stem cell‐based strategy may be applicable to MNDs and other diseases caused by cell loss.

## AUTHOR CONTRIBUTIONS

Min Chen, Xia Wang, and Chuan Li performed experiments, analysed data, and wrote the manuscript. Ting Lan and Yuhui Wei performed animal experiments and collected samples. Chengcheng Tang, Xiaoqing Zhou, Alessandro Rosa, Renping Zhou, Xi Zheng, and Song Ang constructed the plasmids and revised the manuscript. Kun Zhang, Qingjian Zou, and Liangxue Lai designed and supervised the project, and wrote the manuscript.

## CONFLICT OF INTEREST

The authors declare no conflict of interest.

## Supporting information


**Appendix S1** Supporting Information.

## Data Availability

The data that support the findings of this study are available within the manuscript and supplementary materials.
