# Supplementary material for: Inducible motor neuron differentiation of human induced pluripotent stem cells in vivo
Source: Cell Prolif. 2022 Aug 9;55(11):e13319. doi: 10.1111/cpr.13319 (PMC9628231; doi:10.1111/cpr.13319)
Supplement: Supplementary file 1 — Appendix S1 Supporting Information. [file CPR-55-e13319-s001.docx]

**Supplementary Materials and Methods**

**Cell culture**

The hiPSCs line UH10 was a gift from Prof. Guangjin Pan (Guangzhou Institutes of Biomedicine and Health), which maintained on Matrigel-coated (Corning) plates with mTeSR1 medium (Stem Cell) and passaged every 7 days using Accutase (Stem Cell). Cells were cultured at 37 °C, with 5% CO_2_ and 95% humidity. Medium was changed every day. The generation of the UH10 hiPSCs was approved by the Institutional Review Board at Guangzhou Institutes of Biomedicine and Health.

HiPSCs were cultured on Matrigel-coated plates (15–25% confluency) and induced with PSC Neural Induction Medium (Gibco) in accordance with manufacturer’s instructions. After 7 days of neural induction, NPCs (P0) were ready to be harvested and expanded. These induced NPCs were characterized by staining with antibodies against NPCs markers SOX2 and PAX6.

**Generation of stable hiPSCs lines**

HiPSCs were co-transfected with transposable vectors and PiggyBac transposase (PB-*NIL*: transposase = 4:1 or PB-*NIL*: PB-*BLG*: transposase = 2:2:1) using Lipofectamine™ LTX (Invitrogen). After blasticidin (BSD, 10 μg/ml, Invitrogen) selection, the BSD-positive colonies were collected with a pipette and used to generate two stable hiPSC lines (termed *NIL*-hiPSCs and *NILB*-hiPSCs).

**Apoptosis assays**

*NIL*-hiPSCs and *NILB*-hiPSCs were seeded on Matrigel-coated 24-well plate. For the first 24 h after dissociation, cells were treated with 5 μM Rock inhibitor Y-27632 (MCE) to prevent cell death. 3 days after passaging, cells were incubated with 10 μg/ml Hoechst 33258 (Beyotime) and 5 μl Propidium Iodide (PI, Beyotime) for 15 min at room temperature in the dark. Hoechst 33258 and PI fluorescence was observed under Olympus BX51 Microscope. PI positive cells were quantified at 100× magnification. Three non-overlapping fields were randomly selected per well.

**MN differentiation**

*NILB*-hiPSCs were seeded onto Matrigel-coated culture vessels (50,000 per 24-well plate) with or without glass coverslips (NEST). Differentiation was induced by adding 1 μg/ml Dox (Sigma) in MN induction medium as previously described,^1^ which was composed of neurobasal medium (Gibco), 1×B27 (Gibco), 1×Glutamax, 1×NEAA and 0.5×penicillin/streptomycin, and supplemented with 5 μM DAPT (MCE) and 5 μM SU5402 (MCE). Medium was changed every day. After 5 days of Dox induction, neuronal cultures were switched to MN medium supplemented with 10 ng/ml BDNF (Alomone labs), 10 ng/ml GDNF (Alomone labs), 10 ng/ml NT3 (Alomone labs), 1 μM RA (MCE), 0.5 μM Purmorphamine (MCE). The MN medium was composed of neurobasal medium, 1×N2 (Gibco), 1×B27 and 0.5×penicillin/streptomycin.

At 4 weeks of differentiation, cultures were dissociated with Accutase and plated on Matrigel coated wells. 5 μM Y-27632 was added for the first 24 h after dissociation. Neuronal cultures were expanded in MN medium. For further differentiation, neuronal cultures were switched to MN induction medium.

**Isolation and culture of primary neonatal glial cells**

Murine glial cells cultures were prepared from 5-day old wild-type mice as previously described with some minor modifications.^2^ After removing meninges, cortices were dissociated and digested with 0.25% trypsin at 37°C for 15 min. Solution was mixed every 5 min. Dulbecco’s modified Eagle’s medium (DMEM) supplemented with 10% FBS (Gibco), 1×Glutamax, 1×NEAA, 1×Sodium Pyruvate and 1×penicillin/streptomycin was added to the tissue homogenate. After centrifugation at 500 g for 10 min, the cellular pellet was resuspended in DMEM with 10% FBS, 1×Glutamax, 1×NEAA, 1×Sodium Pyruvate and 1×penicillin/streptomycin and then plated on 10-cm dishes. Medium was replaced 48 h after plating. After 1 week incubation, glial cells were passaged and cryopreserved.

**Replating-induced stress**

Murine glial cells were seeded on 24-well plate coated with Matrigel. When the density of glial cells reached 70–80%, a similar number of *NIL*-hiPSCs and *NILB*-hiPSCs were planted on monolayer glia cultures. Dox (1 μg/ml, 5 days) was added to the medium to induce MN differentiation. After differentiation, induced MNs were cultured for another 5 days in MN medium. On day 10, cells were dissociated with Accutase and re-plated on Matrigel-coated 24-well plates. For the first 24 h after dissociation, cells were treated with 5 μM Y-27632 to prevent cell death. Survival rate before replating was set as 100%, to which the subsequent survived cells at each time point were normalized. Images were captured using the Olympus BX51 Microscope. Survived neurons were quantified at 100× magnification. Three non-overlapping fields were randomly selected per well. The number of survived neurons was counted, and the survival rate was calculated for analysis.

**Glutamate toxicity assay**

A similar number of *NIL*-hiPSCs and *NILB*-hiPSCs were planted on Matrigel-coated 24-well plate, respectively. Dox (1 μg/ml, 5 days) was added to the medium to induce MNs. After differentiation, induced MNs were cultured for 7 days without neurotrophic factors. Toxicity was induced by the addition of 20 μM glutamate (MCE) and 100 μM L-trans-Pyrrolidine-2,4-dicarboxylic acid (PDC, Sigma) for 7 days as described.^3^ Cultures were subsequently maintained for additional 7 days for the immunocytochemistry and cell counting.

**Quantitative real-time PCR**

Total RNA was extracted with TRIeasy (Yeasen). About 1.0 μg RNA was reverse transcribed into cDNA with PrimeScript RT reagent Kit (Perfect Real Time) and amplified with SYBR Green PCR Master Mix (Bio-Rad). The internal control used was the housekeeping gene GAPDH. Primer sequences were listed in Supplemental Table S1.

**Immunofluorescence**

Cell cultures at the specified time points were fixed with 4% paraformaldehyde (PFA) for 15 min at room temperature, and then washed with 1×PBS for 4 times. Cells were permeabilized/blocked for 1 h at room temperature in blocking solution (1×PBS containing 0.1% Triton X-100 and 3% BSA). The blocking buffer was removed with no rinsing. Primary antibodies in blocking solution were then added and incubated overnight at 4 °C, followed by washing 4 times with 1×PBS. Fluorophoreconjugated corresponding secondary antibodies in 3% BSA were then added and incubated at room temperature for 1 h in the dark. After incubation, cells were washed 4 times with 1×PBS in the dark. Images were obtained using with Carl Zeiss LSM710 Confocal Microscope or Olympus BX51 Microscope. The following antibodies were used: rabbit anti-PAX6 (Biolegend, 1:200), mouse anti-SOX2 (R&D, 1:200), rabbit anti-OLIG2 (Abcam, 1:200), mouse anti-Isl1/2 (DSHB, 1:200), mouse/rabbit anti-TUJ1 (Abcam, 1:2000), mouse anti-HB9 (DSHB, 1:100), mouse anti-MAP2 (Sigma, 1:200), rabbit anti-ChAT (Abcam, 1:500), rabbit anti-GFP (Abcam, 1:1500), mouse anti-hNuclei (Millipore, 1:400), mouse anti-Ki67 (CST, 1:400), rabbit anti-NANOG (Novus, 1:500), mouse anti-GFAP (Santa, 1:50) and anti-mouse Alexa-488/555 (1:2000, CST), anti-rabbit Alexa-488/555 (1:2000, CST).

**MN differentiation in embryoid bodies (EBs)**

*NILB*-hiPSCs were passaged onto Matrigel-coated plates. When the cells grew to 80% confluence, they were rinsed twice with 1×DPBS and then incubated with Accutase for 3 min. Colonies were gently detached by pipette, and then transferred into 15 ml tube, neutralized with equal volume of EB differentiation medium (Knockout DMEM/F12 + Glutamax + NEAA + 20%KSR), counted, and spun down at 200 g for 4 min. Cells were then re-suspended in EB differentiation medium with Y-27632 (5 μM, 24 h). To form hanging drop EBs, single cell drops (4500 cells/30 μl) were hanging cultured on the lid of Petri dishes, placed in a 37°C incubator. On the second day, EBs formed with uniform size were transferred into U-bottom low-attachment 96-well plates (Thermo). One half of EB differentiation medium was changed daily. Dox (1 μg/ml, 5 days) was added into medium to induce MN differentiation. After Dox induction, EBs were transferred onto Matrigel coated 24-well plates with glass coverslips for immunofluorescence (See the Immunofluorescence as described above). Stained EBs were imaged on a Carl Zeiss LSM710 **(z-stacks** with 1-μm vertical separation of optical sections). Maximum intensity projections were exported in ZEN software. For long-term analysis, EBs (4-week) fixed with 4% PFA were processed and cryo-sectioned for immunofluorescence staining.

**Animal experiments**

SCID immunodeficient mice were purchased from Beijing Vital River Laboratory Animal Technology Co., Ltd. All animal care and experimental procedures are approved by the Ethical Committee on Animal Experiments at Guangzhou Institutes of Biomedicine and Health, Chinese Academy of Sciences.

Teratoma formation. *NILB*-hiPSCs were resuspended in 30% Matrigel in DMEM/F12 (Gibco) containing Y-27632 (10 μM), and then 2× 10^6^ cells in 200 μl Matrigel-DMEM/F12 were injected subcutaneously into SCID mice. Mice were administered i.p. with Dox (25 mg/kg) once daily for 5 days after transplantation. Control mice were injected with PBS. Engraftments were retrieved at different time points (1 w.p.t, 2 w.p.t, 4 w.p.t, and 6 w.p.t.), and fixed in 4% PFA. After fixation, the engraftments were embedded in OCT or paraffin for immunofluorescence staining and H&E staining, respectively.

Four weeks after transplantation, the engraftments were isolated and cultured *in vitro*. In briefly, engraftments were dissociated and digested with 0.05% trypsin at 37°C for 15 min. Solution was mixed every 5 min. Neurobasal medium supplemented with 1×B27, 1×Glutamax, 1×NEAA and 1×penicillin/streptomycin was added to the tissue homogenate. After centrifugation at 500 g for 10 min, the cellular pellet was resuspended in neurobasal medium supplemented with 1×B27, 1×Glutamax, 1×NEAA, 1×penicillin/streptomycin, and then plated on Matrigel-coated wells. For MN differentiation, the cells were shifted to the MN induction medium supplemented with 5 μM DAPT and 5 μM SU5402 in the absence of Dox. After 3 days, the medium was changed to MN medium.

Intra-cerebroventricular injection. *NILB*-hiPSCs were suspended at a final concentration of 1× 10^5^ cells/μl in HBSS (Gibco) containing Y-27632 (10 μM) and Dox (2 μg/ml). The mouse pups (P3–4) were immobilized by cryoanesthesia for 1–2 min on ice. Once cryoanesthetized, 2 μl cells were injected into both lateral ventricles using a Hamilton syringe (1-inch, 32G, Hamilton) as described previously.^4^ 2 days post-transplantation, the pups were administered i.p. with Dox (25 mg/kg, five times) every other day.

Intra-spinal cord injection. *NILB*-hiPSCs were suspended at a final concentration of 1× 10^5^ cells/μl in HBSS containing Y-27632 (10 μM) and Dox (2 μg/ml). The mouse pups (P3–4) were immobilized by cryoanesthesia for 1–2 min on ice. Once cryoanesthetized, the spinal cord was in the midline of the back and it can be seen as a white line. A total of 2× 10^5^ cells dispersed at 2 injection sites of the spinal cord as described previously.^5^ 2 days post-transplantation, the pups were administered i.p. with Dox (25 mg/kg, five times) every other day.

The transplanted mice were killed for immunohistochemistry analysis at 1, 2, 3, or 4 weeks after transplantation. Mice were transcardially perfused with saline followed by 4% PFA fixation buffer, and then their brains or spinal cords were isolated and fixed in 4% PFA overnight at 4°C. After fixation, the brains or spinal cords were cryoprotected in OCT and sectioned at 10 μm on a cryostat (Leica). Immunocytochemistry was performed to assess cell persistence and differentiation. See the Immunofluorescence as described above.

**In vivo bioluminescent imaging**

The survival of transplanted cells was tracked by bioluminescent imaging (IVIS Spectrum). Animals were injected i.p. with substrate D-luciferin (Yeasen), and then anaesthetized with inhaled isoflurane (2–3%). Once anesthetized, mice were imaged for 5 min until the maximum signals were obtained. Bioluminescence signals were quantified in units of maximum photons/sec/cm^2^/steradian of interest region.

**Patch-clamp recordings**

***In vitro*:** The mature induced MNs co-culturing with monolayer glia cells on glass coverslips were put into a recording chamber perfused with bath solution containing (in mM) 140 NaCl, 5 KCl, 2 MgCl_2_, 10 HEPES, 10 glucose, pH adjusted to 7.4 with NaOH. Borosilicate pipettes were filled with a solution containing (in mM) 123 K-gluconate, 10 KCl, 1 MgCl_2_, 10 HEPES, 1 EGTA, 0.1 CaCl_2_, 1 K_2_ATP, 0.2 Na_4_GTP and 4 glucose, pH adjusted to 7.2 with KOH. Neurons were attached by the patch pipette under microscope (Olympus BX51), forming a whole-cell configuration. Whole-cell currents including sodium currents, potassium currents and action potentials were recorded.

***In vivo*:** Mice were anesthetized, spinal cords and brains were removed and quickly immersed in ice-cold oxygenated (95% O_2_ and 5% CO_2_) cutting solution containing (in mM): 50 sucrose, 2.5 KCl, 0.625 CaCl_2_, 1.2 MgCl_2_, 1.25 NaH_2_PO_4_, 25 NaHCO_3_, and 2.5 glucose, pH adjusted to 7.3 with NaOH. Slices (300 mm) were cut using a vibratome (VT 1200S; Leica), and collected in artificial cerebrospinal fluid (ACSF) contained (in mM): 125 NaCl, 2.5 KCl, 2.5 CaCl_2_, 1.2 MgCl_2_, 1.25 NaH_2_PO_4_, 26 NaHCO_3_, and 2.5 glucose (pH adjusted to 7.3 with NaOH). After at least 1 hour of recovery, slices were transferred to a recording chamber and constantly perfused with ACSF (2 ml/min) and visualized under a microscope (Olympus BX50WI). Borosilicate pipettes were filled with a solution containing: 126 K-Gluconate, 4 KCl, 10 HEPES, 4 Mg-ATP, 0.3 Na2-GTP, 10 phosphocreatine, (pH adjusted to 7.2 with KOH). The fluorescence of transplanted *NILB*-hiPSCs-induced cells was examined using a GFP filter. Whole-cell currents including sodium currents, potassium currents and action potentials were recorded.

**Cell counts and quantification**

The percentage of surviving *NILB*-hiPSCs was determined by counting hNuclei^+^ or GFP^+^ cells at cryosections from transplanted animals. *In vivo* conversion efficiency was calculated by the percentage of hNuclei^+^/GFP^+^ cells that expressed neural makers HB9, MAP2, and ChAT from three sections at least, representing the highest number of hNuclei^+^/GFP^+^ cells in each animal. The percentage of double-labeled cells (marker^+^ and hNuclei^+^/GFP^+^) was calculated by dividing the number of double-labeled cells by the total number of hNuclei^+^/GFP^+^ cells.

**Statistical analysis**

All statistics, including statistical tests, sample sizes and types of replicates, are described in the Figure legends. A *P* value of <0.05 was considered to be statistically significant.

**Supplementary Figures and Figure Legends**

**
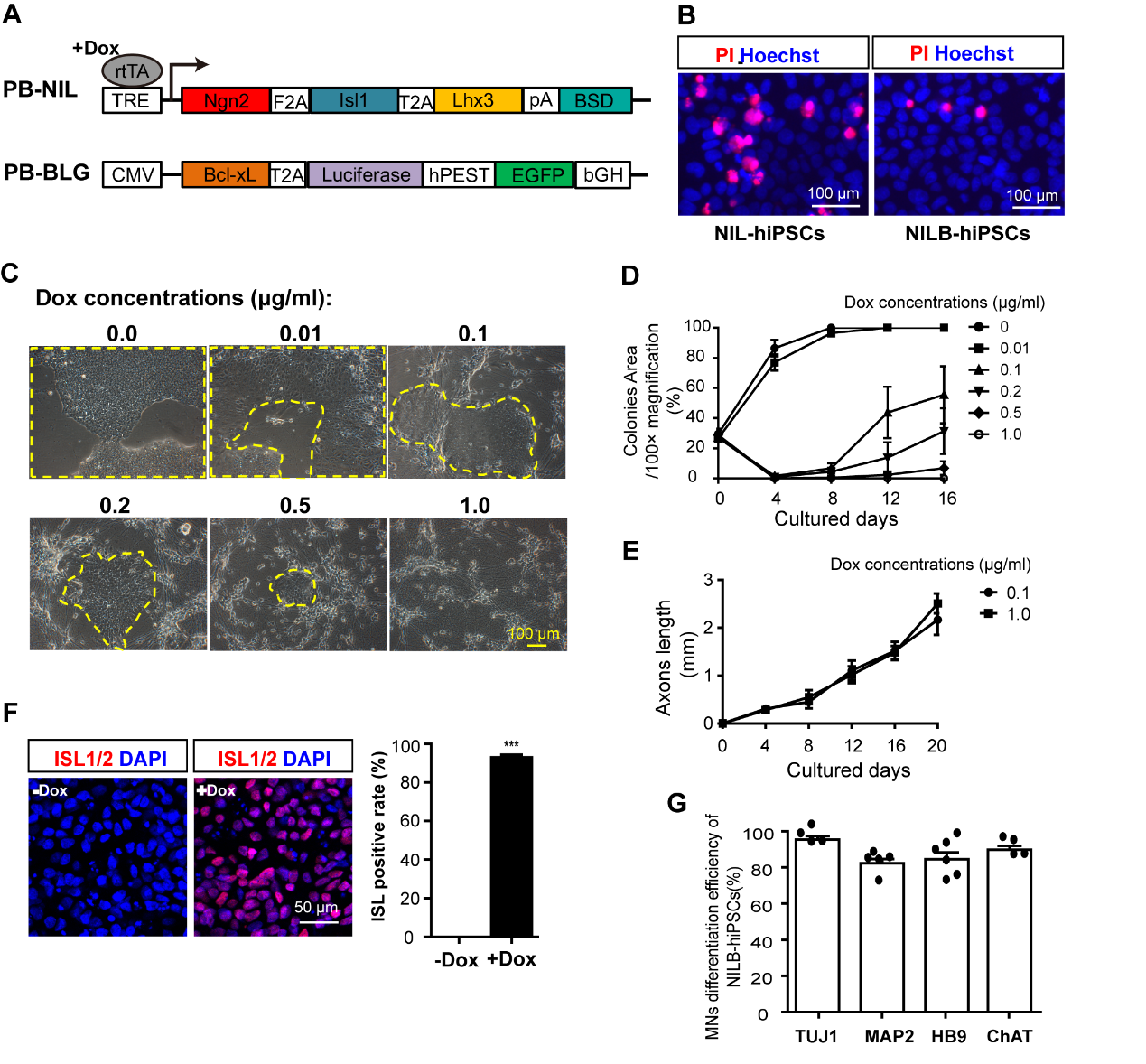
**

**FIGURE S1 Establishment of multi-gene-modified hiPSCs, related to Figure 1.** (A) Structures of PB-*NIL* and PB-*BLG*. (B) Apoptosis analysis of *NIL*-hiPSCs and *NILB*-hiPSCs by PI staining at 3 days after dissociation, bar=100 μm. (C) Undifferentiated *NILB*-hiPSCs colonies (area of yellow dotted line) in MN induction medium with different concentrations of Dox, bar=100 μm. (D) Area ratio of undifferentiated *NILB*-hiPSCs colonies during the neural process with different concentrations of Dox. Error bars represent SEM, n=4 separate differentiation experiments each using the gene modified *NILB*-hiPSCs. (E) Axons length of *NILB*-hiPSCs induced MNs after Dox induction. Error bars represent SEM, n=4 separate differentiation experiments each using the gene modified *NILB*-hiPSCs. (F) Immunostaining for ISL1/2 protein in *NILB*-hiPSCs treated with Dox, bar=50 μm. Error bars represent SEM, ****P*<0.001, n=3 separate differentiation experiments each using the gene modified *NILB*-hiPSCs. (G) Differentiation efficiency of *NILB*-hiPSCs as determined by TUJ1 (n=4), MAP2 (n=5), HB9 (n=6) and ChAT (n=4) expression. Error bars represent SEM. Experiments are independent biological replicates (n).


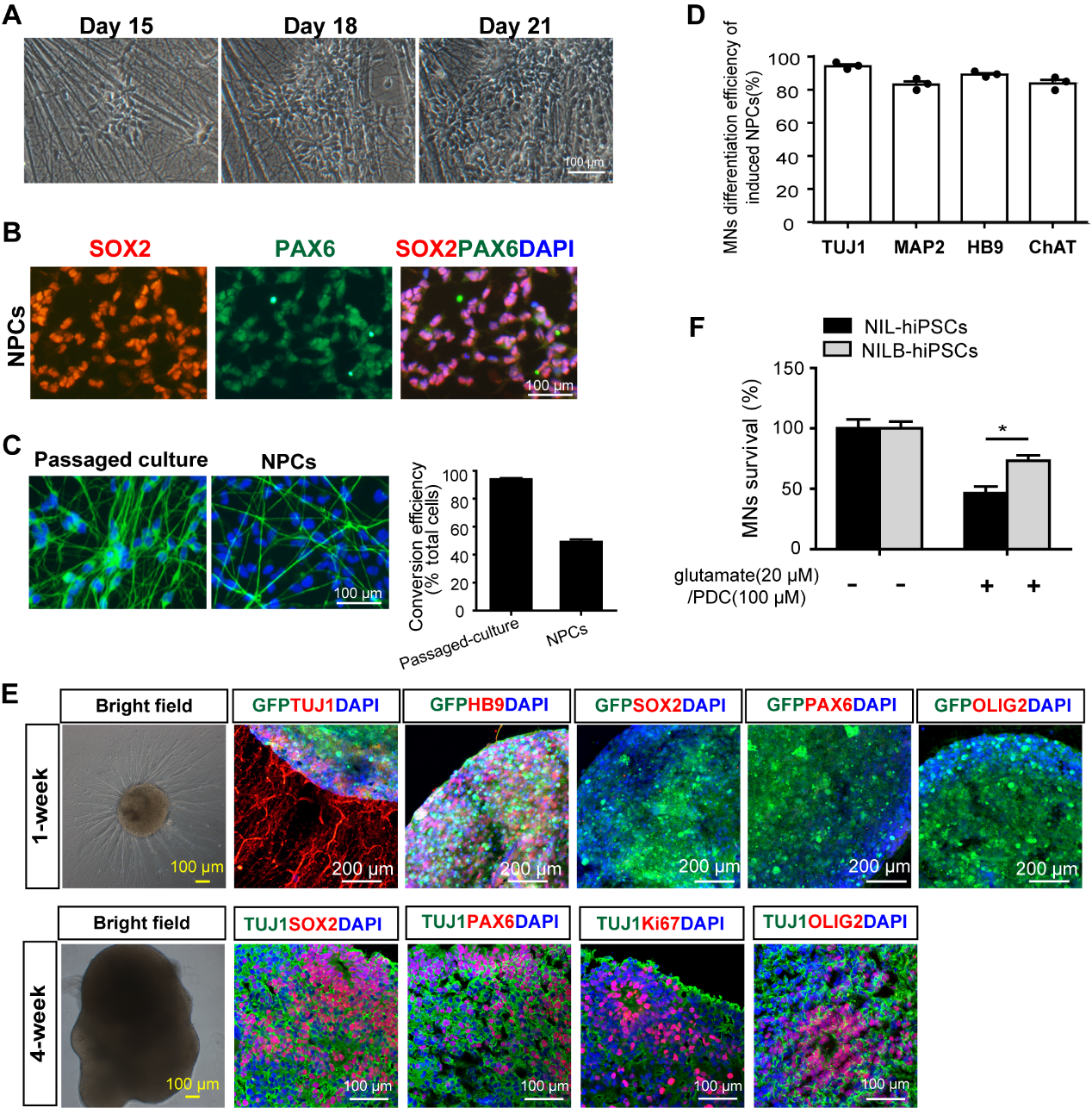


**FIGURE S2 Long time differentiation of multi-gene-modified hiPSCs, related to Figure 2.** (A) Representative images of non-neuronal cells differentiation on day 15, 18, and 21 of MNs induction, bar=100 μm. (B) Immunostaining for the NPCs markers SOX2 and PAX6 in hiPSCs-induced cells, bar=100 μm. (C) Left Panel: Neuronal conversion of passaged-culture and hiPSC-induced NPCs in MN induction medium, bar=100 μm. Right Panel: Conversion efficiency as determined by TUJ1 expression. Error bars represent SEM, n=4 separate differentiation experiments each using the wild-type hiPSCs and gene modified *NILB*-hiPSCs. (D) Re-differentiation efficiency of induced NPCs as determined by TUJ1 (n=3), MAP2 (n=3), HB9 (n=3) and ChAT (n=3) expression. Error bars represent SEM. Experiments are independent biological replicates (n). (E) Upper Panel: Morphology of 1-week EBs after transferred onto Matrigel-coated glass coverslips, bar=100 µm. Immunostaining for neuronal markers (TUJ1 and HB9) and progenitor markers (SOX2, PAX6, and OLIG2) within the 3D EBs, bar=200 µm. Lower Panel: Morphology of 4-week EBs in low-attachment 96-well, bar=100 µm. Immunostaining for neuronal markers (TUJ1) and progenitor markers (SOX2, PAX6, Ki67 and OLIG2) in the 3D EBs cryosections, bar=100 µm. (F) *Bcl-xL* over-expression alleviated glutamate-induced MN toxicity. Error bars represent SEM, **P*<0.05, n=3 separate differentiation experiments each using the gene modified *NIL*-hiPSCs and *NILB*-hiPSCs.


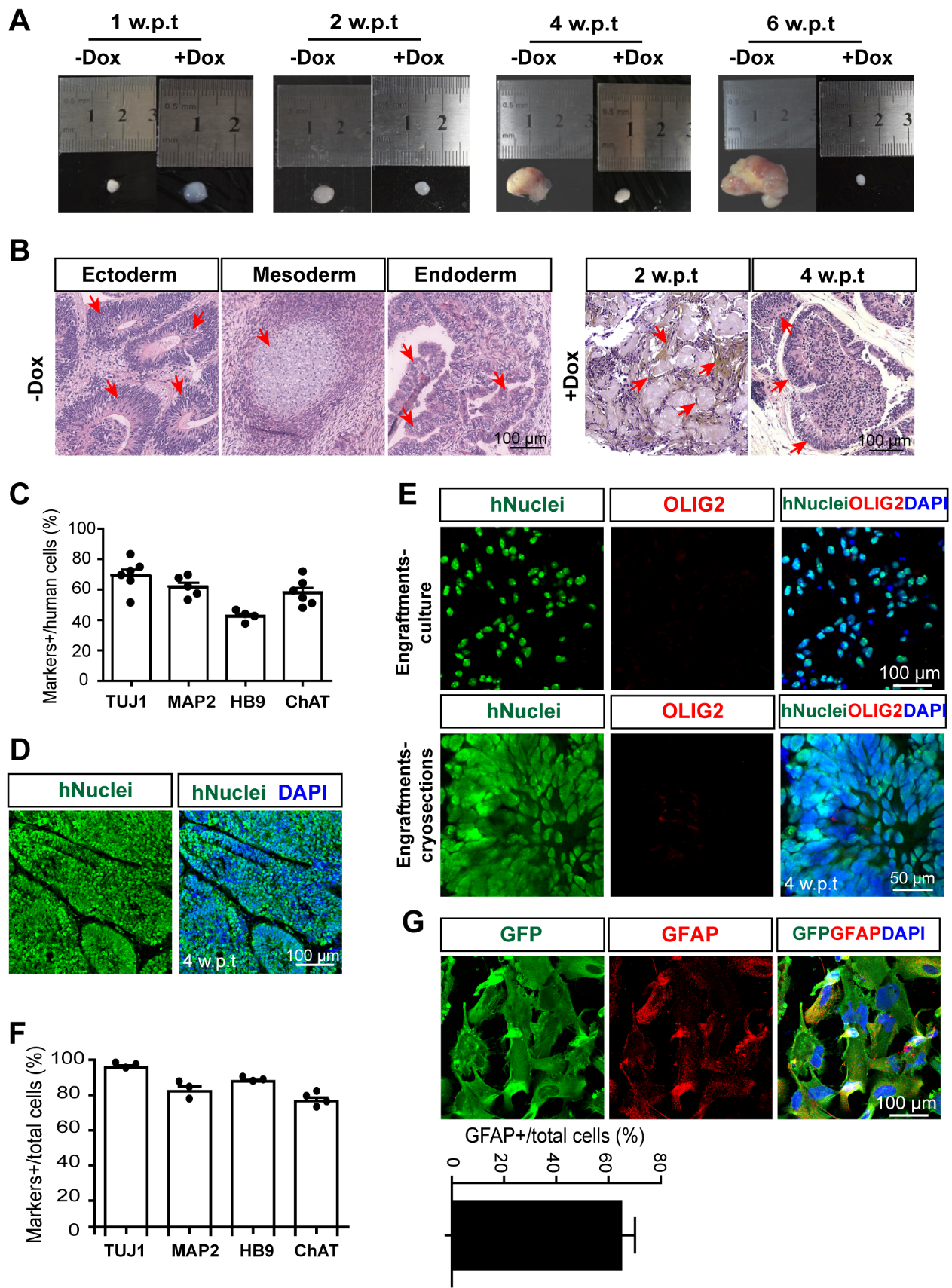


**FIGURE S3 In vivo induction of subcutaneously injected *NILB*-hiPSCs, related to Figure 3.** (A) Visible engraftments formed by *NILB*-hiPSCs after subcutaneous injection with or without Dox treatment. (B) H&E staining of engraftments formed by *NILB*-hiPSCs with or without Dox treatment. Left Panel: development of mature teratoma with identifiable three germ layer derivatives, red arrows indicate neural tube rosettes (ectoderm), cartilage (mesoderm), and ciliated epithelium (endoderm). Right Panel: pyramid neurons (red arrows) and ventricle-like cavities (red arrows) formed by *NILB*-hiPSCs with Dox treatment at 2 w.p.t and 4 w.p.t, respectively, bar=100 μm. (C) Percentages of TUJ1 and hNuclei double-positive cells (n=6), MAP2 and GFP double-positive cells (n=5), HB9 and GFP double-positive cells (n=4), ChAT and hNuclei double-positive cells (n=6) in *NILB*-hiPSCs-transplanted animals. Error bars represent SEM. Experiments are independent biological replicates (n). (D) Massive ventricle-like cavities from 4 weeks post-transplantation animals, bar=100 μm. (E) OLIG2 expression in the engraftments culture (bar=100 μm) and cryosections (bar=50 μm). (F) Percentages of TUJ1 and hNuclei double-positive cells (n=3), MAP2 and GFP double-positive cells (n=3), HB9 and GFP double-positive cells (n=3), ChAT and hNuclei double-positive cells (n=4) in the engraftments-derived MNs. Error bars represent SEM. Experiments are independent biological replicates (n). (G) Glia marker GFAP expression in the engraftments cultured in 10% FBS medium, bar=100 μm. Error bars represent SEM, n=3 separate differentiation experiments each using the gene modified *NILB*-hiPSCs.


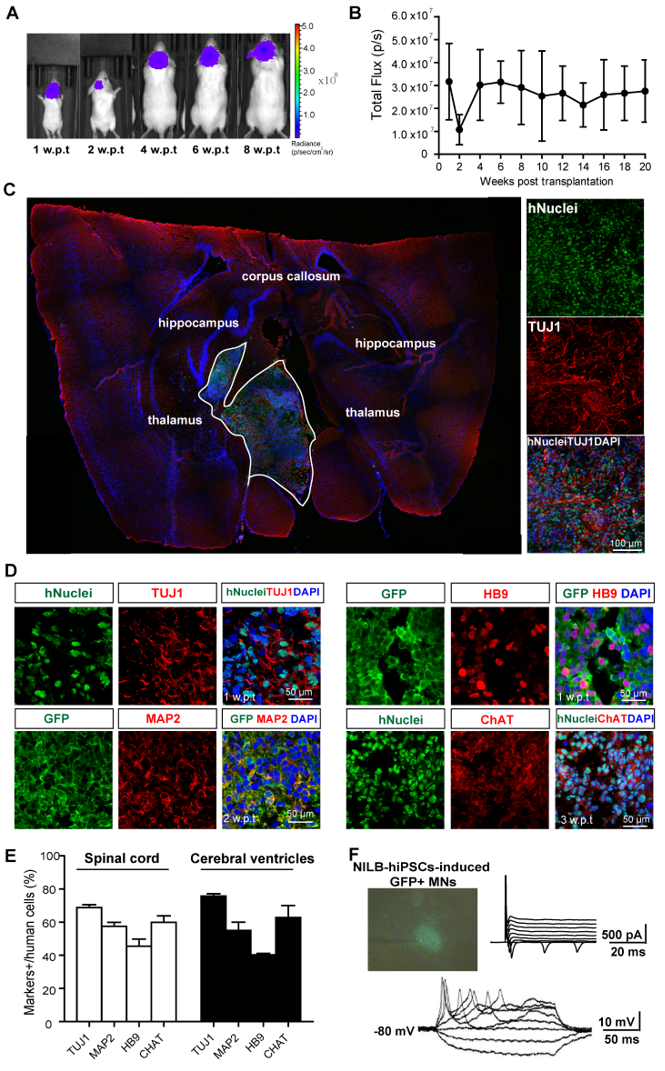


**FIGURE S4 In vivo induction of *NILB*-hiPSCs after intra-cerebroventricular injections, related to Figure 4.** (A) *In vivo* imaging of luciferase activity in the cerebral ventricles of *NILB*-hiPSCs-transplanted animals. (B) Luciferase signal curve of grafts changed over time. Error bars represent SEM, n = 4 separate differentiation experiments each using the gene modified *NILB*-hiPSCs. (C) Representative image of injected *NILB*-hiPSCs in the mouse cerebral ventricles. The transplanted human cells were detected with hNuclei (green) and expressed high levels of TUJ1 (red). Left Panel: Low magnification picture from a coronal section from a transplanted animal. Right Panel: Enlarged images of *NILB*-hiPSCs cells in the cerebral ventricles showing extensive dendritic arborizations after Dox induction, bar=100 μm. (D) Immunostaining for neuronal markers TUJ1, MAP2, HB9 and ChAT in the cerebral ventricles of *NILB*-hiPSCs-transplanted animals, bar=50 μm. (E) Percentages of TUJ1 and hNuclei double-positive cells (n=5), MAP2 and GFP double-positive cells (n=5), HB9 and GFP double-positive cells (n=5), ChAT and hNuclei double-positive cells (n=5) in the spinal cords and cerebral ventricles after transplantation. Error bars represent SEM. Experiments are independent biological replicates (n). (F) Representative traces of action potentials and voltage-dependent ion currents recorded in *NILB*-hiPSCs-derived MNs after intra-cerebroventricular transplantation.


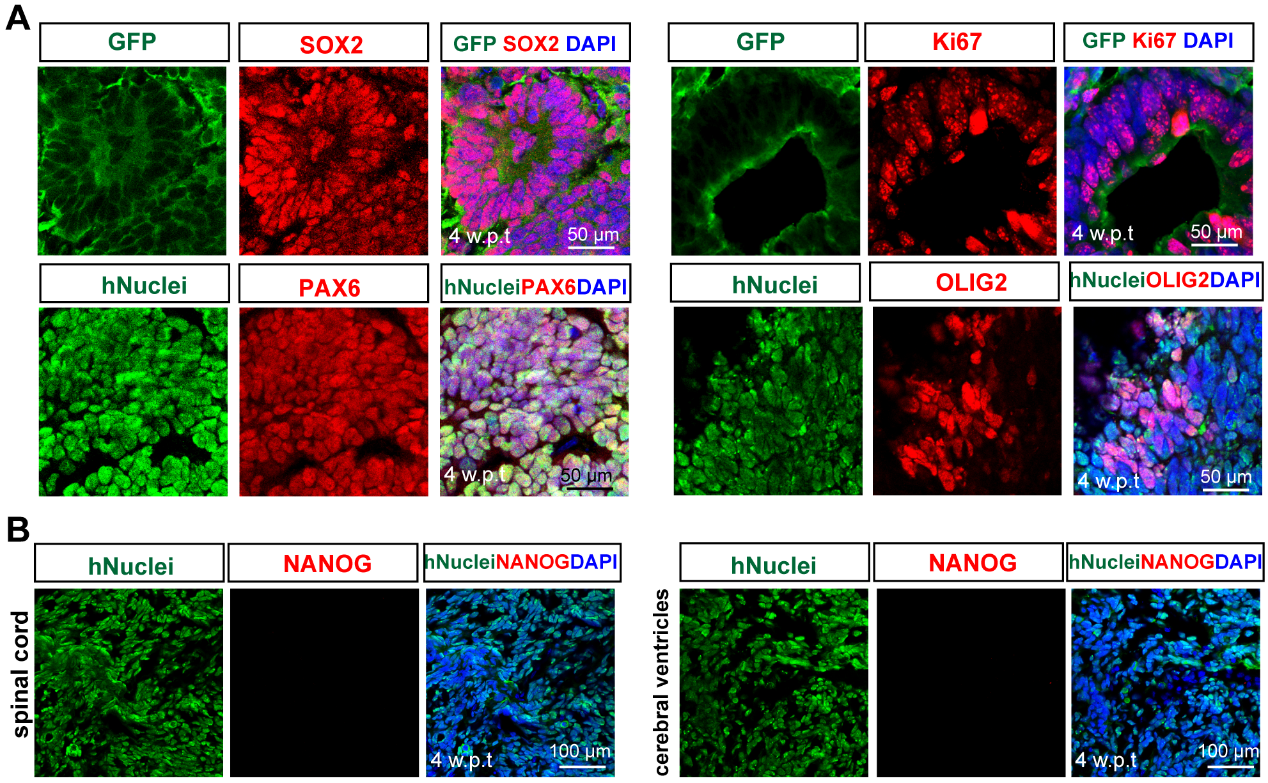


**Figure S5 Induction of NPCs from *NILB*-hiPSCs in the cerebral ventricles of immunodeficient mice, related to Figure 4.** (A) Immunostaining for progenitor markers SOX2, PAX6, Ki67 and OLIG2 in the cerebral ventricles of *NILB*-hiPSCs-transplanted animals, bar=50 μm. (B) Immunostaining for pluripotency marker NANOG in the spinal cord and cerebral ventricles of *NILB*-hiPSCs-transplanted animals, bar=100 μm.

**Table S1 Primers used in this study, related to Figure 1D.**

| Ngn2-F | TATGCACCTCACCTCCCCATAG |
| --- | --- |
| Ngn2-R | GAAGGGAGGAGGGCTCGACT |
| Isl1-F | AAGGTGGAGCTGCATTGGTTTG |
| Isl1-R | TAAACCAGCTACAGGACAGGCC |
| Lhx3-F | ACGGACCCAGTTCTGACCTA |
| Lhx3-R | TGGTCTACCTCATCCAGCCA |
| GAPDH-F | GGAGCGAGATCCCTCCAAAAT |
| GAPDH-R | GGCTGTTGTCATACTTCTCATGG |

**References**

1. De Santis R, Garone MG, Pagani F, de Turris V, Di Angelantonio S, and Rosa A. (2018). Direct conversion of human pluripotent stem cells into cranial motor neurons using a piggybac vector. Stem cell research, *29,* 189-196

2. Vierbuchen T, Ostermeier A, Pang ZP, Kokubu Y, Südhof TC, and Wernig M. (2010). Direct conversion of fibroblasts to functional neurons by defined factors. Nature, *463,* 1035-1041

3. Allodi I, Comley L, Nichterwitz S, Nizzardo M, Simone C, Benitez JA, Cao M, Corti S, and Hedlund E. (2016) Differential neuronal vulnerability identifies IGF-2 as a protective factor in ALS. Sci Rep, *6,* 25960

4. Glascock JJ, Osman EY, Coady TH, Coady TH, Rose FF, Shababi M, and Lorson CL. (2011). Delivery of therapeutic agents through intracerebroventricular (icv) and intravenous (iv) injection in mice. Journal of visualized experiments : JOVE, *56,* 2968

5. Leyton-Jaimes MF, Kahn J, and Israelson A. (2019). AAV2/9-mediated overexpression of MIF inhibits sod1 misfolding, delays disease onset, and extends survival in mouse models of ALS. Proc Natl Acad Sci U S A, *116,* 14755-14760
